# The Role of Psychological and Physiological Factors in Decision Making under Risk and in a Dilemma

**DOI:** 10.3389/fnbeh.2016.00002

**Published:** 2016-01-22

**Authors:** Jonas Fooken, Markus Schaffner

**Affiliations:** ^1^Institute for Health and Consumer Protection, Joint Research CentreIspra, Italy; ^2^Business School, Queensland University of TechnologyBrisbane, QLD, Australia

**Keywords:** risk preferences, elicitation methods, physiological measures, personality traits, dilemma decision

## Abstract

Different methods to elicit risk attitudes of individuals often provide differing results despite a common theory. Reasons for such inconsistencies may be the different influence of underlying factors in risk-taking decisions. In order to evaluate this conjecture, a better understanding of underlying factors across methods and decision contexts is desirable. In this paper we study the difference in result of two different risk elicitation methods by linking estimates of risk attitudes to gender, age, and personality traits, which have been shown to be related. We also investigate the role of these factors during decision-making in a dilemma situation. For these two decision contexts we also investigate the decision-maker's physiological state during the decision, measured by heart rate variability (HRV), which we use as an indicator of emotional involvement. We found that the two elicitation methods provide different individual risk attitude measures which is partly reflected in a different gender effect between the methods. Personality traits explain only relatively little in terms of driving risk attitudes and the difference between methods. We also found that risk taking and the physiological state are related for one of the methods, suggesting that more emotionally involved individuals are more risk averse in the experiment. Finally, we found evidence that personality traits are connected to whether individuals made a decision in the dilemma situation, but risk attitudes and the physiological state were not indicative for the ability to decide in this decision context.

## 1. Introduction

The concept of risk aversion in economics is based on theoretical considerations, although much of its intuition also comes from the daily observation that people avoid taking risks. Both theoretical and common sense understandings of risk aversion are based on the idea that a (stable) underlying individual characteristic reflects that some individuals make more risky choices than others^1^. Besides providing interesting insights for theoretical analysis, risk attitudes have strong implications for many real-life choices. Understanding decision making under risk is therefore of central importance for individuals, businesses and policy makers.

However, it is nontrivial to measure risk attitudes of individuals in an experimental laboratory environment. A large number of studies documents individual-level inconsistencies in experimentally measured risk attitudes (Isaac and James, 2000; Berg et al., 2005; Hey et al., 2009; Dave et al., 2010). Measures obtained from different methods provide conflicting results and can even differ within one method over time (Harrison et al., 2005). However, the drivers of instabilities or inconsistencies of individual risk attitude measures, just as generally the determinants or sources of risk attitudes, are often unclear.

Starting from these observations, we connect risk attitude measures, demographics (age and gender) and personality traits, the physiological state of experimental decision makers and an emotionally stressful trade-off decision when presented with a dilemma. We use elicitation methods by Holt and Laury (HL, 2002, 2005) and Andreoni and Harbaugh (AH, 2009) to measure individual risk attitudes, as they are designed to elicit risk attitudes in the same theoretical framework and employ the same decision variable, which increases comparability. Personality traits are elicited using the *Big Five Inventory* (BFI, Goldberg, 1981; John et al., 2008). In the dilemma situation participants have to decide to save one of two swimmers from drowning after watching a video describing this situation, which is—like risk taking—a potentially emotional decision^2^.

To get physiological data we record the electrocardiogram (ECG) of participants during the decision making process. We focus on heart rate variability (HRV) as a physiological measure, which has been linked to the processing of information in the brain (Critchley et al., 2003) and reflects an individual's sympatho-vagal balance during the decision making process. As such it can inform about the (physiologically reflected) mental activity of individuals at the time of the decision and can link decisions to (potential) emotions. Furthermore, the use of HRV allows to interpret laboratory-based decision making in an out-of-laboratory context, as it allows us to collect objective information without requiring obstructive equipment for our measurements. In the economic literature HRV has been used to study decision making in the context of gambling (Meyer et al., 2000; Wulfert et al., 2005), perceptions of unfair pay (Falk et al., 2011), in stress when being made accountable for decisions (Brandts and Garofalo, 2012), tax compliance (Dulleck et al., 2012), and time preferences (Daly et al., 2009). Fooken (2015) also shows that HRV effects measured in the laboratory are noticeable in terms of out-of-laboratory effects, arguing for external validity. Dulleck et al. (2011) provide general guidelines on linking economic experiments and HRV data. Using physiological data we add to the understanding of factors underlying risk attitudes which have been studied using neuroimaging (Hsu et al., 2005; Kuhnen and Knutson, 2005; Huettel et al., 2006; Platt and Huettel, 2008; Preuschoff et al., 2008; Polezzi et al., 2010), genetic information (Zhong et al., 2009; Carpenter et al., 2011; Dreber et al., 2011) and animal behavior (McCoy and Platt, 2005). The role of emotions on risk taking has also been argued (Loewenstein et al., 2001) and decisions involving risk have been connected to emotions such as anger and anxiety (Gambetti and Giusberti, 2012; Campos-Vazquez and Cuilty, 2014). Similarly, in connection to the dilemma decision we are interested in a better understanding of other emotionally difficult choices.

## 2. Hypotheses

We expect results from our two risk elicitation methods to be correlated, but to provide different estimates of risk attitude of an individual (Dulleck et al., 2015). Our first question is therefore what drives this difference. While drivers of inconsistencies between results from different risk elicitation methods are not fully understood, some factors (i.e., demographics and other personal characteristics) have been shown to influence risk attitudes generally. Most prominent are findings with regard to gender and age. Hartog et al. (2002) connect risk attitudes and demographics; they find that gender and age are related to risk attitudes. Similar results are also included in Halek and Eisenhauer (2001). The experimental literature also suggests that females display more risk aversion, although this result is not always significant (Eckel and Grossman, 2008). While these factors have been shown to matter in some risk elicitation methods, results were not the same across methods. We therefore hypothesize that differences may be based on a more or less pronounced role of gender and age between methods.

Different methods could also reflect risk attitudes in different *risk domains*. If different elicitation methods give more weight to measuring certain domains, personality traits may explain differences between elicitation methods. For example, one method may elicit primarily financial risk taking, another method risk taking in a health and safety context. Thinking of domain-specific risk taking is common in psychological research (Weber et al., 2002), while economists usually consider a more general (underlying) risk attitude across domains, which is nevertheless related to domain-specific risk taking (Dohmen et al., 2011). Personality traits could have a varying influence on risk attitudes in different domains (Soane and Chmiel, 2005). Nicholson et al. (2005) study the connection between personality traits and domain-specific as well as general risk attitudes and find differences between domains. Based on this previous research we hypothesize that risk taking will be positively related to extraversion and openness and negatively related to neuroticism, agreeableness and conscientiousness.

Another source of differences may be the importance of the emotional part in taking risky decisions. We use HRV get a potential correlate of emotional decision-making^3^. HRV has been shown to reflect emotions (Appelhans and Luecken, 2006; Wallentin et al., 2011) and to correlate with emotional states and brain activity (e.g., hapiness, anger, and disgust; see Lane et al., 2009). Another interpretation is that HRV measures mental (and at least partly emotional) stress (Critchley et al., 2003; Gianaros et al., 2004), which may influence risk-taking decisions. Porcelli and Delgado (2009) show that inducing acute stress increases risk aversion in the gain domain and decreases it in the loss domain. Morgado et al. (2015) summarize further literature indicating that inducing stress increases risk aversion. While we cannot define HRV as a measure of acute stress and as our study is somewhat different to the ones mentioned above because we do not induce stress, this could be another channel of how HRV and risk-taking are related. We therefore hypothesize that greater risk taking is related to higher emotional engagement as indicated by HRV. However, if individuals make choices, which are emotionally not too costly, a weak relationship between risk taking decisions and HRV measures is also possible. In this case we may still observe differences across individuals in risk taking, indicating if more or less stressed individuals take higher risks.

With respect to the ability to decide in the dilemma we do not have a clear directional expectation. Emotionally more engaged individuals may be more likely to make the decision of saving one of the swimmers, hesitating less. However, they could also be inhibited in their decision. With respect to other factors we expect more risk averse individuals to be more hesitant, more extrovert individuals to make a decision, and more conscientious and neurotic individuals to hesitate.

## 3. Materials and methods

### 3.1. Heart rate variability measurement

We use information on heart activity of participants to understand their physiological state during the decision making process. Our measurement devices are portable electrocardiogram (ECG) recorders (AR12) with three electrodes attached to a participant's chest. From the recorded ECG we calculate the heart rate variability for a given period. HRV as a physiological indicator is mainly used in medical research (Camm et al., 1996), but also serves as a psychological indicator, whereas the (HRV) ratio $\frac{LF}{HF}$ can be interpreted as psychologically (or emotionally) induced physiological stress (Appelhans and Luecken, 2006). In the absence of major physical activity (such as walking, running, eating etc.) as in a laboratory environment this indicator conveys psychological information (Berntson and Cacioppo, 2008); for example, a higher ratio of sympathetic to parasympathetic activity, which is reflected in the $\frac{LF}{HF}$ ratio (Malik, 2007), has been connected to increased mental stress (Berntson et al., 1994)^4^.

We use heart rate measurements of our participants over the course of the experiment to determine their HRV. These give us a succession of 5 s intervals, which we averaged over the decision time in our analysis (the time between entering and leaving a decision screen). Due to unreadable measurements and instances in which participants made decisions in less than 5 s, data is missing for some choices. Average HRV is comparable across the two risk frameworks. Average HRV during the dilemma was significantly higher than in the two risk frameworks (1.19, *N* = 65, *p* = 0.000). An overview table on HRV measures is also included in the Supplementary Material.

### 3.2. Experimental procedures

We ran our experiment in a computer lab over several sessions on 2 days with a total of *N* = 75 participants. Ethics approval was provided by the QUT University Human Research Ethics Committee (ethicscontact@qut.edu.au) before its start. All participants provided their written informed consent before the start of the experiment. We recruited participants from an online pool of about 2000 students using ORSEE (Greiner, 2015) who had relatively standard characteristics (average age of 21.8 [*SD* = 0.5], 51% male and mostly business end economics students). Our invitation included information about the length of the experiment and that the heart rate of participants would be measured during the study^5^. Upon arrival at the lab, participants were welcomed and asked to put on the heart rate monitor, led to a computer and asked to go through the experiment at their own pace; most participants needed about 30 min to do so. When participants had finished, they were given their payment and returned the heart rate monitor. Participants were paid a show-up fee of five Australian dollars for participating in the experiment plus earnings based on their decisions in the risk elicitation task (on average about 30 Australian dollars).

Our experiment using software CORAL (Schaffner, 2013) had five stages. In the first stage participants were asked personality-related questions of the BFI (John et al., 1991) and other personality-related questions. The second stage included a relaxation phase during which participants were shown a picture of the ocean, heard background sound of the sea rushing on headphones, were asked to close their eyes, take a sea shell from the table into one of their hands^6^, to listen and relax. The relaxation phase lasted for 5 min and aimed to get participants down to an undisturbed baseline heart rate. The following three stages included the two elicitation methods, the dilemma and a demographic questionnaire as described below.

### 3.3. Risk elicitation methods

For both risk elicitation methods, participants were first presented with instructions and had to answer two test questions before advancing to the first round of decisions. Participants played both elicitation methods over two rounds. The method by HL was played first in each round and the method by AH second^7^. For final payments one of the two rounds was randomly selected and from this round one randomly selected choice of each method was determined for final payments to avoid wealth and portfolio-building effects.

In the risk elicitation method by HL, individuals choose between pairs of lotteries. Each pair consists of two lotteries with two options, one with a higher and one with a lower payoff, whereas both lotteries have the same probabilities for the low and high option, but differing dispersion between the outcomes. Participants were presented with a table of nine pairs of lotteries and had to decide for each of these pairs if they prefer the option with more or the one with less dispersion. Going down the table of these nine lottery pairs, the risk premium of choosing the safer lottery (the one with smaller dispersion) increases with every row further down (see HL for further insights on the design of this method and the Supplementary Material for an illustration). As the payoffs remain the same for all nine lottery pairs, individuals chose an optimal switching probability.

The method by AH elicits risk attitudes by letting participants allocate a (convex risk) budget (CRB) between their probability of winning (*prob*) and the amount ω received in case of winning. Each extra percentage point of winning costs the decision maker a certain price (*price*); hence, participants chose their preferred prob^*^ such that their winning amount will be ω^*^ = μ − prob^*^ · *price* with μ being the maximum gain, or budget, that can be won with corresponding *prob*_μ_ = 0. As in this method participants face a direct trade-off between allocating their budget μ to either the probability of winning or the winning amount, given a simple CRRA utility function, a risk aversion parameter can be inferred for each of the 18 decisions taken. In our experiment, participants were informed about the *price* on the top of the computer screen and were able to choose prob^*^ by moving a slider. At the same time they were provided in writing with the corresponding pair of the gain ω^*k*^ in case of winning and the selected *prob*^*k*^. They were also shown a picture of the winning probability in a pie chart and the gain when winning in a bar chart. We refer to the description of the method in AH and the Supplementary Material for instructions, more illustration and an example of this method.

We chose these two methods, as they use a different approach for eliciting risk attitudes, but have been designed with the same theoretical framework in mind (a CRRA utility function) and use the same decision variable (choosing an optimal probability). Furthermore, HL has been widely used in the literature on risk elicitation, connecting our experiment to other studies.

### 3.4. Dilemma decision

After having finished the risk elicitation tasks, participants advanced to the fourth stage that included a video of about 1 min length. The video showed a life saver walking to the beach and then two people drowning in the water. This video was supplemented by a voice asking participants to imagine being in the role of the life saver and having to make a decision of saving one of the two drowning swimmers. Furthermore, it was said that only one of the two could be saved (“you will only be able to save one of them”). At the end of the video, participants automatically advanced to a decision screen that asked them to save either the person on the left or on the right from the video they had just seen. Snapshots of the video showing a hand coming out of the water were included with the choice options. Furthermore, a button for “more information” was included; clicking on this option led to a screen describing more hypothetical options to contemplate about. However, reading this information required time after which both swimmers would have drowned. An option to see even more information (which said that there was no more information) and the option to return to the decision screen were also included^8^.

Participants were given 20 s after entering the decision screen to make a choice and save one of the two swimmers. However, participants were not informed about this time limit and didn't see the clock ticking down. The reason for this was that we wanted to identify those individuals who were able to understand the urgency of the situation and make a decision^9^. If they succeeded in this, they were shown a short video in which the swimmer they had chosen to save was rescued. If they did not make a decision and exceeded the time limit, a time-out screen appeared informing them that they had failed to make a decision.

Finally, participants advanced to a short demographic questionnaire that included information about gender, age, student status and some health related measures (to detect potential problems that could distort heart rate measures), marking the end of the experiment.

## 4. Results

### 4.1. Analysis separated by methods

**Figure 1:**
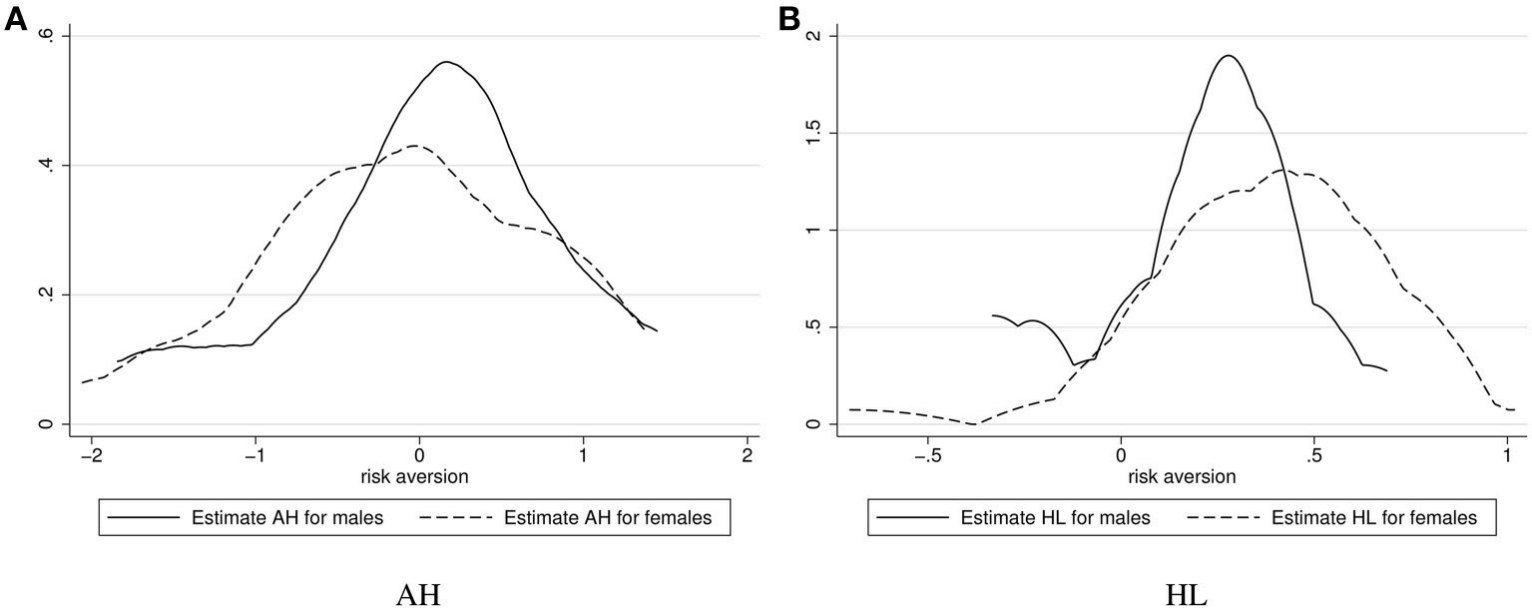
**Distributions of *r*_*i*_-parameters estimated using the two methods separated by gender**. **(A)** AH. **(B)** HL.

We used two methods for the elicitation of risk attitudes, providing data that allows us to compare results across methods. We estimated individual-specific risk aversion coefficients *r*_*i*_ assuming $U_{i}\left(x\right)=x^{1-r_{i}}$ in an expected-utility (EUT) framework for each method^10^. (For AH it is even possible to determine a coefficient of risk aversion *r*_*it*_ for every choice). This allows us to get an idea about the distribution of individual risk attitudes for both methods and make some general comparisons between them. Figures 1A,B illustrate the estimated values for those participants with *SD*(*r*_*it*_) ≤ 3 for AH and participants with less than 4 switching points in HL^11^.

As can be seen in these overviews, there seem to be differences between the distributions of individual estimates by gender. Women seem to be more risk loving than men in the AH method, but more risk averse in the HL method, the second of which is in line with previous findings (Eckel and Grossman, 2008). Individual values for the methods, $r_{i}^{AH}$ and $r_{i}^{HL}$, are significantly correlated at 0.53 for males (*N* = 31, *p* = 0.002; Spearman's ρ = 0.40, *N* = 31, *p* = 0.025), 0.41 for females (*N* = 29, *p* = 0.027; ρ = 0.50, *N* = 29, *p* = 0.005), and 0.42 for both males and females together (*N* = 60, *p* = 0.001; ρ = 0.38, *N* = 60, *p* = 0.002).

In order to investigate a relationship between gender and age, and risk attitudes, we included these in our method-specific estimation procedure^12^. We also tested for a relationship between risk attitudes and some of the personality traits and HRV measurements. In both methods we used maximum likelihood estimations for this^13^. Table 1 reports the results of this procedure.

**Table 1:** **Determinants of *r*-estimates for AH and HL**.

	**AH1**	**AH2**	**AH3**	**HL1**	**HL2**	**HL3**
*r*	0.24^**^	0.04	0.16	0.41^***^	0.35^***^	0.43^***^
	(0.10)	(0.55)	(0.61)	(0.03)	(0.11)	(0.09)
Female		−0.10	0.14		0.16^***^	0.13^**^
		(0.20)	(0.22)		(0.05)	(0.06)
Age		0.01	0.00		−0.00	−0.01^*^
		(0.03)	(0.03)		(0.00)	(0.00)
Extraversion			0.00			−0.04^*^
			(0.08)			(0.03)
Agreeableness			−0.02			−0.04^*^
			(0.12)			(0.03)
Conscientiousness			−0.06			−0.01
			(0.10)			(0.03)
Neuroticism			−0.08			−0.01
			(0.13)			(0.02)
Openness			−0.14			−0.02
			(0.12)			(0.03)
HRV ($\frac{LF}{HF}$)			0.23^*^			0.02
			(0.13)			(0.05)
N (individuals)	66	66	58	64	64	57
n (choices)	1188	1188	1044	1152	1152	1026

*** indicates significance at the 1% level,

** 5% significance, and

* 10% significance.

Standard errors (in brackets) are clustered by individuals. The HRV measure represents average HRV during the AH stage in AH_3_ and average HRV during the HL stage in HL_3_.

The results from the estimation support the first impression that the role of gender is different between the two methods: There is no apparent relationship between gender and risk-taking in AH and a significant relationship in HL, indicating higher risk aversion in females (0.16, *N* = 64, *p* = 0.003). Similarly, there is no age effect in the AH method and an economically small effect in the HL method (−0.01, *N* = 57, *p* = 0.086).

Adding personality characteristics and HRV measures does not change the significance level of any of the variables in the AH method and reports the personality characteristics as insignificant. However, there is a significant relationship (−0.23, *N* = 58, *p* = 0.088) between *r*^*AH*^ and the HRV ($\frac{LF}{HF}$), indicating that individuals who were physiologically more stressed displayed more risk aversion in the AH method. This effect is also visible when correlating $r_{i}^{AH}$ and HRV_*i*_ for this period (−0.21, *N* = 60, *p* = 0.099).

In the HL method adding information on personality traits shows that some of the personality characteristics are significantly related to risk attitudes: There is a positive connection to extraversion (−0.04, *N* = 57, *p* = 0.095) and agreeableness (−0.04, *N* = 57, *p* = 0.098). For extraversion, this is as expected. For agreeableness, the result is in the opposite of our expectation, which was based on previous findings in the literature. Furthermore, there is no clear evidence that for the HL method HRV is significantly related to risk taking (−0.02, *N* = 57, *p* = 0.703) and the same is visible when correlating $r_{i}^{HL}$ and HRV_*i*_ (0.13, *N* = 60, *p* = 0.340)^14^.

While this physiological measure of the HRV is insignificant for the HL method, the relationship is significant for the AH method^15^. This difference could be due to the fact that the level of risk taking in HL is relatively stable between the choices, while the risk taken between AH choices can vary noticeably: In HL for a slightly risk averse individual the first and last three rows of the choice list might be straightforward, while only the pivotal ones are critical in a physiologically relevant way. For AH in each period a full range of risky and riskless options can be chosen and most individuals make both risk-seeking and risk-averse choices during the course of this method. Hence, individuals vary more in their level of risk taking and deviations from a potential stress optimal decision can be detected in the data^16^ ^17^. Another possible explanation is that the two methods measure different types of risk taking, which could also be reflected in the difference of their estimated values and their connection to demographics and personality traits, whereas one is simply more strongly related to emotions and therefore more strongly reflected in the physiological state of the decision maker than the other.

### 4.2. Analysis of jointly estimated values for *r* and of drivers of potential differences between HL and AH measures

As the methods by HL and AH were designed with a utility function of $U_{i}\left(x\right)=x^{1-r_{i}}$ in mind, *r*_*i*_ can be estimated with data from both methods in a joint procedure. Assuming this joint structure, we investigated which variables had a significant influence on the jointly estimated risk attitude *r*_*i*_. Table 2 illustrates different specifications, showing that there is no variable with a significant impact on *r*. While this could indicate that these variables simply have no significant connection to the risk attitudes, another reason could be that the two methods are measuring (slightly) different things, and the joint estimation washes out some of the effects visible in Table 1.

**Table 2:** **Determinants of the joint estimation assuming no structural difference between the methods**.

	**JOI_11_**	**JOI_12_**	**JOI_13_**	**JOI_14_**	**JOI_15_**
*r*	0.28^***^	0.00	0.14	0.27^***^	0.14
	(0.06)	(0.40)	(0.33)	(0.08)	(0.31)
Female		0.00	0.15		0.14
		(0.14)	(0.14)		(0.14)
Age		0.01	0.00		0.00
		(0.02)	(0.01)		(0.02)
Extraversion			−0.04	−0.05	
			(0.06)	(0.06)	
Agreeableness			−0.02	−0.05	
			(0.08)	(0.07)	
Conscientiousness			−0.05	−0.06	
			(0.07)	(0.08)	
Neuroticism			−0.02	−0.02	
			(0.06)	(0.06)	
Openness			−0.07	−0.06	
			(0.08)	(0.08)	
HRV ($\frac{LF}{HF}$)			0.15	0.12	0.15
			(0.09)	(0.10)	(0.10)
N (individuals)	60	60	54	54	56
n (choices)	2160	2160	1944	1944	2016

*** indicates significance at the 1% level,

^**^5% significance, and ^*^10 % significance. Standard errors (in brackets) are clustered by individuals.

We therefore investigated differences between the two methods in estimates for *r*_*i*_ and potential determinants of such differences. We did so assuming *U*(*x*) = *x*^1−*r*+Δ*HL*^ with Δ*HL* representing the difference between the methods (AH was used as the baseline and Δ*HL* hence reflects the additional effect of HL). We estimated *r* and Δ*HL* to identify potential determinants of differences in measured risk attitudes. However, we cannot find a significant difference between the methods except for extraversion which plays a significant role in having a higher risk attitude in HL compared to AH (0.12, *N* = 54, *p* = 0.058). Hence, there is an unclear difference between the methods, potentially with any possible difference being blurred by a too large measurement noise in the data. The results from our estimations are included in the Supplementary Material.

### 4.3. Determinants making a timely decision

We used the life saving dilemma to investigate how gender, age, risk attitudes, personality traits, and physiological states during the decision process were related to the ability of individuals to save a swimmer. Table 3 shows results of probit regressions of making a decision to save one of the swimmers (or exceeding the time limit otherwise).

**Table 3:** **Probit regressions of decision to save swimmer**.

	**SLS_1_**	**SLS_2_**	**SLS_3_**	**SLS_4_**
Female	−0.05	−0.15	0.23	0.13
	(0.30)	(0.38)	(0.36)	(0.44)
Age	−0.00	0.01	0.00	0.01
	(0.03)	(0.03)	(0.03)	(0.04)
*r*_*i*_(JOI_11_)		0.18	0.16	0.10
		(0.29)	(0.27)	(0.33)
Extraversion		−0.28		−0.61^***^
		(0.19)		(0.24)
Agreeableness		−0.21		0.13
		(0.21)		(0.26)
Conscientiousness		−0.34^*^		−0.51^**^
		(0.19)		(0.24)
Neuroticism		−0.38^*^		−0.49^**^
		(0.22)		(0.24)
Openness		0.04		0.25
		(0.20)		(0.25)
HRV ($\frac{LF}{HF}$)			0.00	0.06
			(0.18)	(0.19)
constant	−0.27	−0.70	−0.64	−0.90
	(0.59)	(0.77)	(0.65)	(0.84)
N (individuals)	71	61	59	56
n (choices)	71	61	59	56

*** indicates significance at the 1% level,

** 5% significance, and

* 10 % significance.

Gender and age had no significant effect on the ability to make a decision. A similar conclusion is true for risk attitudes (using the joint estimate of an individual's risk attitude *r*_*i*_). This is somewhat surprising, as we conjectured a more risk averse decision maker to be more hesitant. In this sense risk attitudes measured in the laboratory do not link to this dilemma decision-making aimed to detect a hesitating character. Personality traits had an influence on the decision to save one of the swimmers although their sign is not always as expected (in specification SLS_2_: conscientiousness −0.34, *N* = 61, *p* = 0.072; neuroticism −0.38, *N* = 61, *p* = 0.093; in specification SLS_4_: extraversion −0.61, *N* = 56, *p* = 0.011; conscientiousness −0.51, *N* = 56, *p* = 0.032; neuroticism −0.49, *N* = 56, *p* = 0.040). However, it seems that individuals with moderate personality traits are more likely to make a decision. Finally, there was no clear relationship between the physiological activity of the 20 s during which the decision had to be made and the ability to make a decision. The same is true using simple correlations or Spearman's ρ to investigate a correlation. This indicates that being emotionally more stressed is not related to the ability to make this decision in time.

## 5. Discussion

Our aim was to understand how risk attitudes, measured using two different elicitation methods, were linked to demographics, personality traits and the physiological state at the time choices were made. We also wanted to link these latent factors of the decision making process to the ability to make a decision in a dilemma. We find that there seems to be a shifter effect in risk attitude measures between the methods; women are significantly more risk averse in HL but not in AH. However, there is no statistically significant general and also no significant gender effect driving differences between the two methods. For personality traits there is only evidence that more extraversion and agreeableness are related to more risk seeking in HL, while there is no significant effect in AH. Again there is however no clear indication that personality traits drive the difference between the methods. We interpret this such that domain-specific risk taking, which prior literature showed to vary with personality traits, is no major determinant of differences in risk attitude measures between the two methods.

We also investigated how risk attitudes and physiological states were connected, using HRV data as an indicator for mental stress during the time of decision making. We find that HRV and risk taking are related in a way that individuals who show lower physiological responses during the decision take higher risks in one of the methods (AH). One interpretation of this is that emotionally less stressed individuals take more risk. This suggests that risk taking is something not just momentarily stressful but reflected in a more basic physiological state. Hence, when emotionally less involved (potentially stressed) individuals take more risky decisions, this (physiologically) reflects their general attitude toward risk while making the decision, rather than their immediate reaction to the risk task at hand. This could for example mean that the emotional part in risk taking has a more gradual effect than a prompt emotional reaction which would be indicated in an immediate bodily response. Although the effect was not significant in the other method (HL), the observed relationship to HRV does not seem to be just an artifact of one method, as the direction of the effect is the same for HL and there is a significant effect in HL when allowing for probability weighting.

Finally, we linked risk attitudes, demographics, personality traits and physiological states to the ability to make a timely decision in a dilemma. We find that, except for some of the personality traits (extraversion, conscientiousness, and neuroticism) these were not significantly related to making the decision to save one of the swimmers. Hence, if emotions are connected to decision making in the dilemma and this emotional part is reflected in HRV, this would indicate that higher emotional involvement does not influence the ability to make a timely decision in our dilemma. We conclude with the finding that individuals who are less emotionally involved as indicated in their physiological state during the experiment were also less risk averse, while the ability to make a dilemma decision was not significantly linked to the physiological state, but rather personality-based.

### 5.1. Further interpretations

Both findings are interesting for their implications in real life, for example in companies in which risky (financial) decisions are taken by individuals. If more emotionally stressed individuals are more risk averse, this might be worth considering when finding employees who take decisions involving high risks for these organizations or their customers. However, this interpretation is partly speculative and further research is needed, as our study is only one piece of research and cannot answer if within-individual deviations in experimental risk attitudes are reflected in different physiological states between different days or if our results mainly reflect differences between individuals. Either result could be worth considering, for example when managing personnel in companies, selecting certain individuals for specific positions, or for creating (more or less emotionally stressful) work environments.

With respect to the decision in the dilemma our results indicate that it is more the underlying personality which leads to the ability to decide, while the emotional involvement of this situation is less important. This could be an interesting consideration, for example when selecting and managing employees in jobs where dilemma-like decisions have to be made, for example in emergency rooms. However, again further research would need to substantiate our findings before making recommendations to management. Nevertheless, our results can, despite their explorative nature, be interesting for individual and institutional decision makers for whom understanding emotional decision making processes is a major factor for personal and organizational success.

### 5.2. Limitations

Some limitations of the study should also be acknowledged. One first is that we study decision making in a laboratory environment, which can only partially represent decision making in daily life. However, they may give some indications of underlying mechanisms in risk-taking decisions, which are also important outside of the laboratory.

A second limitation can be seen in the correlational nature of our analysis. That is, while giving first indications, we are not able to pin down the causal relationship between risk taking and emotions. A third point is that we use HRV as an indicator of emotions. While theoretical considerations under the risk-as-feelings hypothesis as well as previous research on HRV make it very reasonable to interpret it as reflecting emotional decision making, without knowing which exact emotions are at work (which we cannot determine with our given data), our results stand as one piece of evidence which should be supported by further studies.

## Author contributions

The authors contributed equally to all parts of the research.

### Conflict of interest statement

The authors declare that the research was conducted in the absence of any commercial or financial relationships that could be construed as a potential conflict of interest.

## Footnotes

^1^The term *risk aversion* is usually used with an expected utility (EUT) paradigm in mind, where the curvature of the utility function can be understood as a measure of risk aversion. We will rely on EUT, although more advanced theories exist (e.g., prospect theory, PT or rank-dependent utility theory, RDU, see Wakker, 2010). Interpreting risk attitudes using these theories can be *richer* than under EUT, but they often require richer data. We address the connection to other theories if our data allows us to and keep these in footnotes for interested readers.

^2^Dilemmas are traditionally investigated in philosophical thought experiments. The dilemma we use is similar to the *doctor's dilemma* or a more benign version of *Sophie's Choice* (Greenspan, 1983) in which one person's life can be saved at the cost of another person's life. Marcus (1980) and Greenspan (1983) discuss emotions and the feeling of guilt that are involved when deciding in a dilemma. Emotional engagement in dilemma decisions is also emphasized in the study of neural correlates connected to moral judgements in dilemmas (Greene et al., 2001), which finds that emotions play an important role in a (dilemma-related) decision making process.

^3^We do not hypothesize that HRV is related to decisions such that it explains behavior; rather, it correlates with and reflects mental states. Using *reverse inference*, we interpret the fact that decisions and changes in the physiological state occur at the same time such that they are causally related. Using reverse inference in neuroeconomic research is common, but not unproblematic (Phelps, 2009). For the potentially causal link between HRV and mental activity see below.

^4^Interpretations of HRV measures mainly rest on the understanding that the autonomous nervous system (ANS) is influenced by the sympathetic and parasympathetic systems and that the influence of the two systems is reflected in the heart rate. Other systems are active alongside, regulating e.g., respiration, body temperature, and blood pressure; the influence of these other systems is eliminated in our HRV data. The sympathetic system is responsible for fight-or-flight responses, using sympathetic nerves and hormones (particularly adrenaline). The parasympathetic system controls rest and relaxation through specific pacemaker cells. Both systems are constantly active parallel to each other, but the degree to which one of the systems controls the heart rate in a given period varies. The two systems operate at different speeds. Changes in the heart rate due to increased sympathetic activity have a longer time horizon compared to parasympathetic activity. Increases in sympathetic activity have their strongest effect between 5 and 20 s, increases in parasympathetic activity after less than 5 s. This allows for a decomposition of the heart rate into different frequencies that allow to observe the varying importance of sympathetic and parasympathetic activity. Practically, this is done in estimation procedures using waves of different lengths (or frequencies). Using decompositions into frequencies and studying their relative influence (power) at a given time allows to identify the effect of the sympathetic and the parasympathetic system and mediates some of the individual heterogeneity in heart rate data. More information on the estimation procedures is available upon request. The ratio of the low frequency (LF, 0.033–0.15 Hz) to the high frequency (HF, 0.15–0.4 Hz) mirrors the activity of sympathetic to parasympathetic activity.

^5^No explicit exclusion criteria were used and our post-experimental questionnaire which included questions on health conditions also did not indicate any obvious exclusions.

^6^The Purpose of the sea shell was to prevent participants from crossing their arms and distorting the heart rate measurement by interfering with the electrodes on their chest.

^7^There should be no order effect between the methods (Dulleck et al., 2015).

^8^This screen only served the purpose of using up participant's time. Many participants clicked one or even both “more information” buttons, and some of these still managed to save one of the two swimmers.

^9^That is, we did not want to study the stressful effect of having to decide under time pressure, which might affect the decision (Kocher et al., 2013), but the effect of emotions on the propensity to make a timely decision.

^10^We extended our analysis beyond EUT for HL assuming a probability weighting function $w_{i}\left(p\right)=\frac{p^{\gamma_{i}}}{{\left(p^{\gamma_{i}}+{\left(1-p\right)}^{\gamma_{i}}\right)}^{1∕\gamma_{i}}}$. We estimated ${\bar{r}}_{i}=$ .04 (*SD* = 0.34) and ${\bar{\gamma }}_{i}=$ 1.16 (*SD* = 0.14) using HL. Hence, there is some evidence for probability weighting, however, we cannot determine a γ_*i*_ significantly different from 1 (representing no probability weighting) for most participants, probably due to our relatively small sample. See the Supplementary Material for distributional overviews and more detail.

^11^In AH this leads to the exclusion of five individuals and in HL of six individuals. We use these restrictions as *r*_*i*_-estimates for these individuals do not appear very meaningful in the context we are using; making these exclusions does not qualitatively change the overall distribution, but makes the graphs more readable.

^12^Throughout this paper we use normalized values for all variables in our estimations.

^13^See Harrison and Rutström (2008) for a guideline on estimation procedures. Here likelihood functions used were $L_{HL}=\prod_{i=1}^{18}\left[Pr{\left(y_{i}|X_{i},\alpha ,\beta \right)}^{y_{i}}\cdot {\left(1-Pr\left(y_{i}|X_{i},\alpha ,\beta \right)\right)}^{1-y_{i}}\right]$ for HL with *y*_*i*_ = 0 if option A was chosen, *y*_*i*_ = 1 if option B was chosen and assuming *Pr*(*y*_*i*_ = 1|*X*_*i*_, α, β) reflected in a normal distribution and $L_{AH}=\prod_{i=1}^{18}\left[\frac{prob_{k}|X_{i}^{\alpha }}{\overset{maxprob}{\underset{j=1}{\sum }}prob_{j}|X_{i}^{\alpha }}\right]$ for AH with *prob*_*k*_ indicating the probability chosen by the decision maker and *prob*_*j*_ ∈ [1, …, *maxprob*] indicating all probabilities not chosen. For joint estimations in the analysis described further below we used $L_{joint}={\left(L_{HL}\right)}^{d}\cdot {\left(L_{AH}\right)}^{1-d}$ with *d* = 1 for HL observations and *d* = 0 for AH observations.

^14^An extension of our analysis allowing for probability weighting in HL (see Supplementary Material) shows a significant relationship between estimates of γ and HRV and also a potential relationship for *r* in HL. Nevertheless, this result is not as easily interpretable, as *r* and γ are jointly needed to determine an individual's risk attitude. However, the result can be seen as indicating that the connection between HRV and risk attitudes is influenced by probability weighting, whereas more stressed individuals are more likely to tend toward *inverse S shape*-type probability weighting.

^15^Generally, both have the same direction. The sign of this relationship was visible throughout most alternative specifications we looked at.

^16^Indeed, there is evidence that in the AH method in periods where individuals have a lower $\frac{LF}{HF}$ they take more risky choices. However, this effect is not significant.

^17^This interpretation may also be supported by observing the result from including the variance of HRV instead of HRV in the estimation, which is even more statistically significant than the HRV variable itself for AH. However, it is again not significant for HL.
