# Supplementary material for: The Role of Psychological and Physiological Factors in Decision Making under Risk and in a Dilemma
Source: Front Behav Neurosci. 2016 Jan 22;10:2. doi: 10.3389/fnbeh.2016.00002 (PMC4722108; doi:10.3389/fnbeh.2016.00002)
Supplement: Supplementary file 1 [file DataSheet1.pdf]

Supplementary Material: The role of psychological and physiological factors in decision making under risk and in a dilemma

Jonas Fooken* and Markus Schaffner
*Correspondence:
Jonas Fooken
jonas.fooken@gmail.com

1 HRV OVERVIEWS

Supplementary Table 1. Summary statistics of HRV ($\frac{LF}{HF}$) during the 3 decision frameworks

	Measure	Mean	Stand. Dev.	Min.	Max.
All Risk (AH+HL)	$\frac{LF}{HF}_{all}$	.202	1.49	-1.18	12.69
	$\frac{LF}{HF}_{indiv.means}$	.130	1.18	-1.47	3.61
AH	$\frac{LF}{HF}_{all}$	.178	1.52	-1.16	12.69
	$\frac{LF}{HF}_{indiv.means}$	.087	1.14	-1.43	3.78
HL	$\frac{LF}{HF}_{all}$	.269	1.41	-1.18	9.97
	$\frac{LF}{HF}_{indiv.means}$	.160	1.04	-1.21	4.13
Dilemma	$\frac{LF}{HF}_{all}$	.017	1.12	-1.16	8.58
	$\frac{LF}{HF}_{indiv.means}$	1.722	1.23	.13	5.59

The table shows summary statistics of HRV measures during the decision frameworks. $\frac{LF}{HF}_{all}$ summarizes the HRV of all observations during these periods, $\frac{LF}{HF}_{indiv.means}$ the HRV measures averaged by framework for each individual (the second of which was used for the analysis).

2 DIFFERENCES BETWEEN THE TWO METHODS

Supplementary Table 2. Determinants of the differences between the two methods

	JOI_{21}	JOI_{22}	JOI_{23}
r	0.28***	0.28***	0.29***
	(0.04)	(0.05)	(0.05)
ΔHL	0.02	0.35	0.13
	(0.07)	(0.39)	(0.27)
Female		-0.17	-0.16
		(0.11)	(0.13)
Age		-0.01	0.00
		(0.02)	(0.01)
Extraversion			0.12*
			(0.06)
Agreeableness			0.05
			(0.07)
Conscientiousness			0.03
			(0.06)
Neuroticism			0.05
			(0.08)
Openness			0.01
			(0.06)
HRV ($\frac{LF}{HF}$)			-0.07
			(0.07)
$\Delta\frac{LF}{HF}$			-0.05
			(0.07)
N (individuals)	57	57	54
n (choices)	2052	2052	1944

*** indicates significance at the 1% level, ** 5% significance and * 10 % significance. Standard errors are in brackets. Including demographic, personality trait and physiological variables in JOI_{21-23} to explain r provide no further insights, but makes the table less readable. $\Delta\frac{LF}{HF}$ measures $\frac{LF}{HF}(AH) - \frac{LF}{HF}(HL)$. For JOI_{23} 4 participants older than 30 were excluded for the estimation model to converge. Leaving out the (insignificant) age variable and including these 4 participants provides the same qualitative results.

3 EXTENSION BEYOND EUT

We also looked at results beyond an EUT framework by allowing for probability weighting and assuming a utility function of $U_i(x,p) = w_i(p) \cdot v_i(x)$ where $w_i(p)$ represents a probability weighting function, assigning objective probabilities a subjective weight, and $v_i(x)$ the utility from receiving outcomes. We used functional forms of $w_i(p) = \frac{p^{\gamma_i}}{(p^{\gamma_i}+(1-p)^{\gamma_i})^{1/\gamma_i}}$ and $v_i(x) = x^{1-r_i}$.

Supplementary Figures 1 (a) and (b) illustrate the distributions of individual r_i and γ_i estimates for the HL method.[1] For this overview and in the following analysis we have restricted our sample to 45 individuals that had a single switching point (SSP) in both periods of HL to make the graphs readable and to ensure convergence of our maximum likelihood models.

[1] For AH no estimates of γ_i and r_i can be found, as the set of possible choices in each decision is too large (30 choice options of which the optiomal one is chosen).

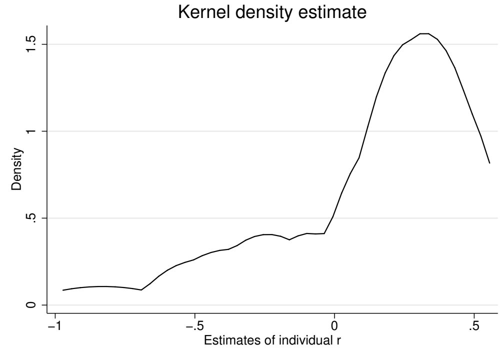

(a) Distribution of r

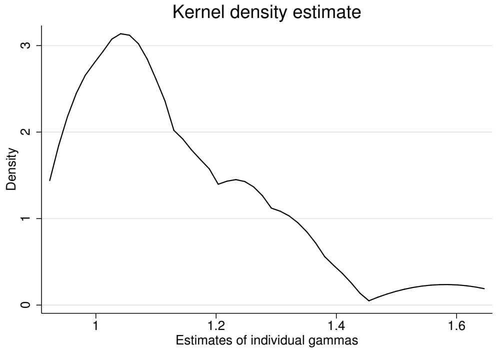

(b) Distribution of γ

Supplementary Figure 1. Distributions of r_i- and γ_i-parameters estimated using HL

We further investigated how demographics, personality traits and physiological states were related to r and γ. Supplementary Table 3 includes the results from our analysis, showing potential personality and gender effects for r and γ, respectively, which are however not robust to alternative specifications. The same is true for the role of HRV in r. However, there is a significant relationship between HRV and probability weighting suggesting that more stressed individuals are more likely to display *inverse S shape*-type probability weighting. This could indicate that the relationship observable before in the EUT-based analysis could be driven by probability weighting.

Supplementary Table 3. Determinants of r and γ

	REDU1	REDU2	REDU3	REDU4
r	0.27***	0.31***	0.29***	0.35***
	(0.06)	(0.07)	(0.02)	(0.08)
Age	0.00	-0.00		-0.00
	(0.00)	(0.00)		(0.00)
Female	0.05	0.02		-0.02
	(0.04)	(0.05)		(0.05)
Extraversion		-0.06		-0.07**
		(0.05)		(0.03)
Agreeableness		-0.02		-0.02
		(0.03)		(0.03)
Conscientiousness		0.02		0.01
		(0.04)		(0.03)
Neuroticism		-0.00		-0.01
		(0.02)		(0.01)
Openness		0.01		0.01
		(0.02)		(0.02)
HRV ($\frac{LF}{HF}$)			-0.04	-0.06*
			(0.03)	(0.03)
γ	1.06	0.94	1.05	1.04
	(0.03)	(0.11)	(0.03)	(0.13)
Age	-0.00	0.01		0.01
	(0.00)	(0.01)		(0.01)
Female	-0.02	-0.10		-0.28**
	(0.03)	(0.16)		(0.13)
Extraversion		-0.07		-0.10
		(0.18)		(0.08)
Agreeableness		0.06		0.05
		(0.06)		(0.05)
Conscientiousness		0.04		-0.01
		(0.14)		(0.04)
Neuroticism		-0.03		-0.02
		(0.06)		(0.03)
Openness		-0.04		0.00
		(0.04)		(0.04)
HRV ($\frac{LF}{HF}$)			-0.11***	-0.18***
			(0.02)	(0.06)
N (individuals)	45	43	42	40
n (choices)	810	774	756	720

*** indicates significance at the 1% level, ** 5% significance and * 10 % significance. Standard errors (in brackets) are clustered by individuals. The availability of HRV data has reduced the sample between estimations (due to missing or unreadable data).

4 INSTRUCTIONS AND DETAILS ON THE RISK ELICITATION METHODS

4.1 Introduction

The following part, or game, of this session is an economic experiment. This means that the amount of your final payment will depend on the decisions you take in the following stages. I.e., your decisions taken on the next screens, together with the random outcome of an external probability distribution, will directly translate into how much you will be paid at the end of the experiment. Please follow the instructions carefully, and please raise your hand if you have a question: an experiment administrator will come to you. During the experiment, any talking or other communication between participants is forbidden.

You will make decisions during this experiment by responding to questions displayed on the computer screen in front of you. After you have completed your responses for the decisions on each screen, please press the Continue button at the bottom of the screen to proceed to the next screen. Your decisions in this experiment are anonymous, and you are identified solely by your participant number. The payment you will receive at the end of the experiment will be kept confidential from all other participants.

This experimental game will be continued over two rounds. You will receive instructions for each step of the experimental game on your screen. At the conclusion of the experiment, the computer will randomly select one decision from Type One and one decision from Type Two to be played to determine the amount that you will be paid. This means that you (or the administrator) do not know which decision will be selected. Therefore, it would be reasonable to treat each decision as if it were the decision that will be selected for determining your final payoff.

4.2 HL instructions

4.2.1 HL example instructions

Please make sure to read all instructions very carefully. This is an instruction screen. You do not have to make any decisions on this screen. On the NEXT screen you will have to make nine decisions between two lotteries. For each of the nine decisions you MUST select either option A or option B. Each lottery is characterized by the probability of receiving one of two payoffs. Below is an EXAMPLE of only two of the nine decisions that you will be required to make

Option A 1					Option B 1			
p	X_A	*1-p*	Y_A		*p*	X_B	*1-p*	Y_B
0.3	3	0.7	1		0.5	5	0.5	0

In decision 1 you have to choose between lottery A1 and lottery B1. In lottery A1 you either receive $1 with probability 0.3 and $3 with probability 0.7. In Lottery B1 you get $0 with probability 0.5 and $5 with probability 0.5.

Remember, at the end of the experiment one decision will be selected at random. This decision will then be played out and will then contribute to your final payment. Because the decision that is played is selected randomly you do not know which decision will be selected and hence it would be reasonable to answer all decisions as if they were the decision that determined your final payment. When you select an option, an X will indicate your choice. You can revise your choice as many times as you like. After you have made all nine choices, click the Continue button to move to the next screen.

4.2.2 HL choice list

Supplementary Table 4 shows the choice list table presented to experimental subjects.

Supplementary Table 4. Multiple price list design as in HL

Option A				Option B			
p	X_A	*1-p*	Y_A	p	X_B	*1-p*	Y_B
0.1	8	0.9	6.4	0.1	15.4	0.9	0.4
0.2	8	0.8	6.4	0.2	15.4	0.8	0.4
0.3	8	0.7	6.4	0.3	15.4	0.7	0.4
0.4	8	0.6	6.4	0.4	15.4	0.6	0.4
0.5	8	0.5	6.4	0.5	15.4	0.5	0.4
0.6	8	0.4	6.4	0.6	15.4	0.4	0.4
0.7	8	0.3	6.4	0.7	15.4	0.3	0.4
0.8	8	0.2	6.4	0.8	15.4	0.2	0.4
0.9	8	0.1	6.4	0.9	15.4	0.1	0.4

The table represents the lottery choice for $X_A = 8, Y_A = 6.4, X_B = 15.4$ and $Y_B = .4$ used in the first round. In the second round we used lotteries with $X_A = 10, Y_A = 8, X_B = 19.25, Y_B = .5$.

4.3 AH instructions

4.3.1 AH example instructions

In this part of the experiment you will consider many options of gambles. The gambles will differ according to the amount of money at stake and the chances of winning that money. An option of gambles might look like this. Notice, you see all available gambles in the option by moving the slider bar back and forth, GIVE IT A TRY! The pie chart represents the probability of winning while the bar chart represents the possible gain. See how there is a tradeoff between these two variables as you move the slider.

Maximum gain is \$10.00. Each 1 percent increase in the pie decreases possible earnings by \$0.10. Each 1 percent decrease in the pie increases possible earnings by \$0.10

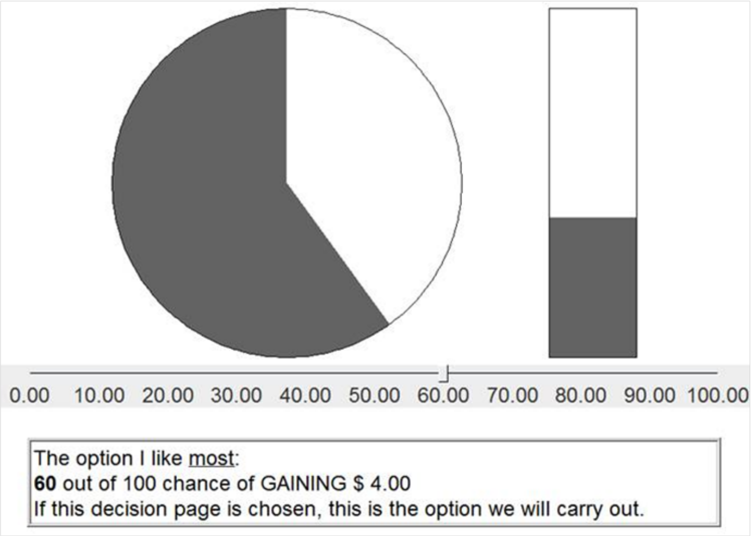

Supplementary Figure 2. AH picture

Notice that in this example, every time you try to increase the chance of winning by 1 percentage point, you reduce the amount you would gain by \$0.10. Likewise, each time you increase the amount you can gain by \$1, you reduce the chance of you winning it by 10 percentage points (that is 1 divided by 10). You are simply required to position the slider in the position that you like the most for each of the nine decision screens. Just as before, only one of your nine decisions will be selected at random. Because you

do not know which decision will be selected it would be reasonable to make each decision as if it were the decision that contributed to you final payment.

4.3.2 AH choice pairs

Supplementary Table 5 includes the 9 pairs of budgets μ and $price$ in the two rounds of our experiment.

Supplementary Table 5. Pairs of maximum gain and cost of probability

Round	1	2	3	4	5	6	7	8	9
μ	27.3	56	172	88	49.4	39.2	54.5	207	116
$price$	0.28	1.17	10.75	2.75	0.77	0.41	0.68	8.62	2.42

The $price$ reflects the cost of getting 1% extra of a winning probability, and μ the amount that can be won with a corresponding probability of zero, or the budget. To win with any positive probability, participants have to buy additional winning probability. For example, in round 1, a participant could chose to win $27.3 - 10 \cdot 0.28 = 24.5$ with a probability of 10%, $27.3 - 20 \cdot 0.28 = 21.7$ with a probability of 20%, and so on.

5 EXAMPLES OF EXPERIMENTAL SCREENS

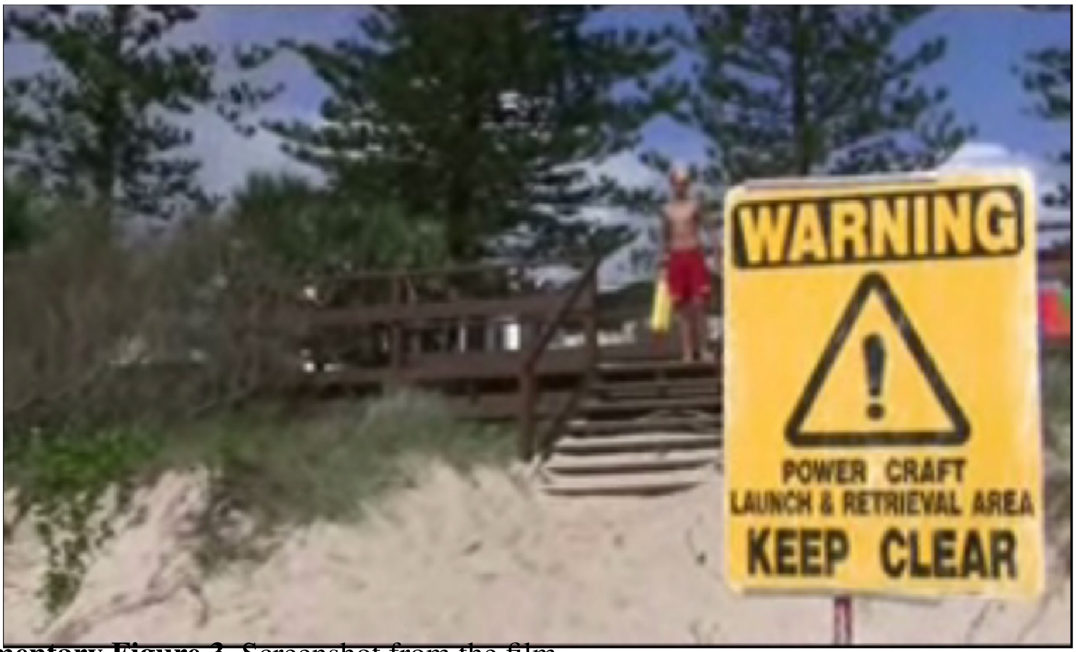

Supplementary Figure 3. Screenshot from the film

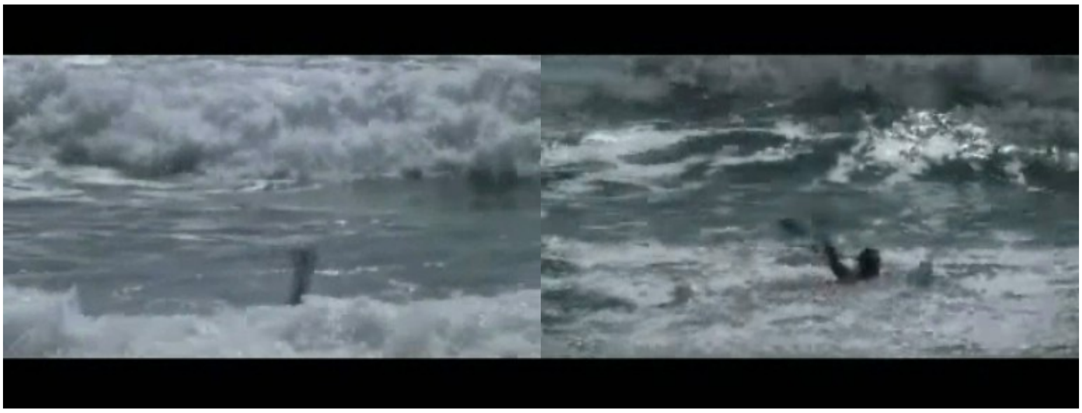

(a) Save left swimmer (b) Save right swimmer

Supplementary Figure 4. Pictures shown for option to save one of the two swimmers
